# Associations Among Sleep, Emotional Eating, and Body Dissatisfaction in Adolescents

**DOI:** 10.1007/s10578-024-01692-4

**Published:** 2024-04-05

**Authors:** Megan L. White, Olivia M. Triplett, Nuria Morales, Tori R. Van Dyk

**Affiliations:** Department of Psychology, Loma Linda University, 11130 Anderson Street, Suite 106, Loma Linda, CA 92350 USA

**Keywords:** Adolescents, Sleep difficulties, Emotional eating, Body dissatisfaction

## Abstract

The literature on adolescent sleep has shown a bidirectional relationship between sleep difficulties and altered eating habits, including emotional eating. However, it is unclear if this relationship is related to preexisting body concerns, or if poor sleep is the prime contributor to emotional eating patterns. This study therefore seeks to examine body dissatisfaction as a moderator of the sleep-emotional eating relationship in an at-risk sample. Adolescents (*N* = 106) presenting for overnight polysomnography self-reported on time-in-bed, insomnia, body dissatisfaction, and emotional eating. Less time-in-bed was correlated with a greater desire for thinness and greater insomnia severity was related to overall emotional eating and eating in response to anxiety, anger, and frustration and in response to depression. Moderation analyses revealed that the relationships between time-in-bed and eating in response to feeling unsettled (*b* =  −.002, 95% CI[− .003,  − .001], *p* < .005) and eating in response to anxiety, anger, and frustration (*b* =  −.01, 95% CI[− .01,  − .001], *p* < .05) were exacerbated by worse body dissatisfaction. Optimizing sleep may attenuate the risk for disordered eating, particularly for adolescents with high body dissatisfaction.

Sleep difficulties (i.e., difficulty falling asleep, staying asleep, or insufficient sleep) have become an increasing problem for adolescents [12]. Such sleep difficulties are associated with negative outcomes such as worsened mood [38], poor emotion regulation [36], and compensatory changes in eating behavior [46]. While the literature on the relationship between sleep difficulties and mood (i.e., depression and anxiety) is substantial, minimal research has been conducted on the relationship between sleep difficulties and emotion-driven eating. Furthermore, little is known about the association between sleep difficulties and a negatively skewed view of self (i.e., body dissatisfaction). Considering the onset of disordered eating has the highest prevalence in teens between the ages of 14 and 19 years of age [47], understanding the relationships among sleep difficulties, emotional eating, and body dissatisfaction is particularly crucial for informing adolescent physical and mental health care.

Approximately 80% of adolescents in the United States regularly sleep less than the recommended eight hours per night [11], sleeping approximately three fewer hours per night during the school week than during the weekend [48]. This is concerning as short sleep in adolescence is associated with worse emotional and behavioral functioning. Sleep deprivation is shown to disrupt prefrontal lobe maturation, as well as the cortical functioning circuit in the adolescent brain, thus impairing adolescents’ executive functioning and inhibitory control [33]. With decreased performance in this region, sleep-deprived adolescents are more likely to experience poor emotion regulation and increased behavioral impulses [33]. Further, short sleep is associated with higher sensitivity to the negative affect of others, higher likelihood of experiencing negative emotions, and reduced expression and feeling of positive emotions [38]. For example, one experimental study found habitual sleep insufficiency to be related to lower positive affect and an increased propensity toward negative moods in response to challenges and stress [17]. Taken together, adolescents experiencing poor sleep may be at increased risk for pronounced anger, risk-seeking behavior, and depressed affect.Sleep-deprived adolescents may also be more prone to utilize coping behaviors that are chosen based on hedonistic needs (i.e., pleasure, attention, indulgence) rather than rational, homeostatic needs. Such hedonistic coping is often seen in food intake changes [13]. Adolescents reporting fewer hours of sleep simultaneously report higher food-related disinhibition traits that may exacerbate food-seeking behavior, especially for palatable (i.e., high sugar, high salt, high fat) foods [7]. One experimental study demonstrated that moderately restricted sleep (i.e., 6.5 h per night) led teens to double their intake of sweet foods compared to when obtaining healthy sleep [5]. Other studies have examined sleep timing and variability and eating behavior in youth, showing that shorter weekday sleep duration is associated with higher eating in the absence of hunger (EAH), while greater weekend “catch up” sleep is associated with lower EAH [27]. Further, Parker et al. [39] found shorter sleep duration, earlier sleep midpoint, and later wake times to be associated with greater loss-of-control eating, further supporting that sleep duration and timing may be linked to eating behavior.

Despite previous research findings, mechanisms underlying the relationship between sleep and eating disturbances remain largely unknown [19]. Increased food reward, emotional reactivity, decreased inhibitory control, and metabolic disturbances have been suggested as factors that may augment the likelihood of increased energy intake among sleep-disturbed adolescents [19]. Molecular mechanisms related to sleep and appetite regulation have also been implicated, such as associations between total sleep time reduction and decrease concentrations of leptin, an appeitite hormone related to satiety, and increased concentrations of ghrelin, an appetite-stimulating hormone [27, 46]. This relationship has been seen in children as young as six months of age, with infants enduring sleep difficulties, including inability to sleep, having more irregular food intake patterns even 2–4 years later [30].

Insomnia and sleep duration in children and adults have also been linked to alterations in eating patterns, such as eating at irregular hours and/or eating quickly [50] and higher rates of external and emotional eating [9]. While eating in the absence of hunger and loss of control eating have been previously examined within the context of sleep, that relationship between emotional eating and sleep in adolescents is minimal. Emotional eating refers to the tendency to eat in response to emotional triggers as opposed to a true physiological need for food [2]. While there are numerous triggers leading to emotional eating, researchers have hypothesized that before emotional eating takes place, individuals experience some form of negative emotionality that they feel unable to control and subsequently compensate with a controlled behavior: eating [21]. Emotional eating appears to occur in response to psychological symptoms such as depression, anxiety, and loneliness – all of which create negative emotionality in varying degrees [20]. As the need to regulate negative emotionality heightens, the desire to eat may become maladaptive, leading to emotion-triggered overeating.

Considering the bidirectional relationship between sleep difficulties and emotional distress [52], it is possible that individuals with impairments in their sleep quality may also experience high levels of dysregulated and impulsive eating as a means of emotion regulation [42]. An experimental study of children aged 5–12 years revealed that decreased sleep duration was associated with higher levels of external eating (i.e., eating in response to the palatable appearance of food), and decreased sleep continuity was associated with increased emotional eating, or food intake in response to emotional distress [9] While the connection between sleep difficulties and emotional eating has been less researched in adolescents, the literature suggests that eating highly palatable foods in response to emotional distress is highly probable in this population [44]. As previously mentioned, emotional eating often occurs in response to depression, anxiety, and loneliness [20], which are frequently observed in adolescence [51]. Therefore, adolescents experiencing sleep difficulties may also experience emotional stress, which may increase the risk of emotional eating [42].

Furthermore, emotional eating may be linked to the internalization of culturally prescribed body images, and consequently, higher rates of body dissatisfaction. Previous studies have found numerous cultural, social, physical, and psychological changes that negatively affect body image for both adolescent males and females [53]. For example, the changes that occur during puberty are among the most impactful in human development, encompassing rapid changes in weight, body shape, and sex characteristics. These changes often coincide with heightened media exposure as well as subsequent comparisons to cultural ideals of beauty. For many young males and females, lean and muscular bodies as well as ultra-thin bodies, respectively, tend to be idolized [45]. Prior research suggests a relationship between higher levels of body dissatisfaction and increased emotional eating among adolescents [26] and adults [16, 57]. Among adolescents who are overweight/obese, higher body dissatisfaction is predictive of increased emotional eating, although not among normal-weight teens [43]. This may be because body dissatisfaction causes negative feelings related to one’s body and/or appearance [8], which may trigger eating as a way of coping with negative feelings.

Further, poor sleep and body dissatisfaction may also be related, although less research exists in this area. Preliminary examinations suggest that sleep may play a role in the perception and judgments of a person’s own view of their attractiveness and health, or their body satisfaction [3]. In females aged 18–22, attempts to decrease body weight, fears of becoming fat, binge-eating episodes, and higher levels of body image dissatisfaction have been shown to be significantly associated with sleep maintenance difficulties and non-restorative sleep [46]. The association between body image dissatisfaction and sleep disturbances may be attributed to the negative, self-critical thinking that often results from body image dissatisfaction, which in turn may negatively impact sleep [23]. Adult-focused studies have found that insomnia symptoms are significantly associated with greater dissatisfaction with cutaneous body image (i.e., the mental perception of the appearance of one’s own skin, hair, and nails; [1]. However, there is limited research on the relationship between poor sleep and dissatisfaction with body shape and size in adolescents.

In sum, insufficient sleep among adolescents is common and presents a significant health concern. Due to greater sleep deprivation [4], impulsivity [33], high sensitivity to negative stimuli [31], and increased risk for body dissatisfaction associated with puberty-related body changes and social pressure, adolescents may be at the highest risk for emotional eating as a means of emotion-regulation [41]. Further, greater impulsivity, compensatory eating fluctuations, anxiety, and depression have all been shown to covary with decreased sleep in adolescents [15]. Thus, adolescents may be at a greater risk of developing a clinical eating disorder as these maladaptive patterns worsen without professional attention [56]. However, limited research exists on the association of sleep difficulties in adolescence with body dissatisfaction and emotional eating.

The current study will attempt to fill these gaps by examining the relationships between sleep disruption (i.e., time-in-bed and insomnia severity), body dissatisfaction, and emotional eating, as well as the interaction between sleep disruption and body dissatisfaction on emotional eating among a sample of adolescents presenting for an overnight sleep study. Adolescents with pre-existing sleep concerns were selected based on their increased risk for general emotion dysregulation, eating in response to emotional dysregulation, and increased body dissatisfaction. Based on prior research, it was hypothesized that less time-in-bed and greater insomnia severity would be related to greater emotional eating and body dissatisfaction, and that greater emotional eating and body dissatisfaction would be related. Further, it was hypothesized that there would be a moderating effect of body dissatisfaction on the relationship between sleep disruption and the urge to engage in emotional eating such that greater body dissatisfaction would exacerbate the relation between sleep disruption and emotional eating.

## Method

### Participants

Participants included 106 adolescents ages 12–18, presenting to the Loma Linda University Health Sleep Disorders Center (LLUH-SDC) for polysomnography (PSG) to assess for a sleep disturbance. Participants were part of a larger study examining the relationship between sleep, mood, behavior, and physical health among a sample of youth with sleep concerns. For the current study, exclusion criteria included participants who were not accompanied by a parent/legal guardian, those who were unable to read and speak English, and those who were not between the ages of 12–18 years.

### Procedure

Participant recruitment at the LLUH-SDC occurred from August 2019 to March 2020 and from January 2022 to June 2023, with a temporary pause due to the COVID-19 pandemic. Upon presentation to LLUH-SDC for an overnight PSG, sleep technologists asked families if they wanted to hear about a research study focused on pediatric sleep and behavior. If families agreed, a trained research assistant described the study in more detail. After obtaining informed parental consent and child assent from interested families, the youth and their parent/legal guardian were given tablets to independently complete the survey questionnaire. Given the clinical setting and number of constructs measured, attempts to reduce participant questionnaire burden were made (e.g., utilizing short-form measures). Families were given an opportunity to enter an Amazon gift card raffle ranging from $25 to $100. This study was performed in line with the principles of the Declaration of Helsinki and all procedures were approved by the Loma Linda University Institutional Review Boards.

### Measures

#### Demographic Information

Parents/legal guardians reported demographic information about their child including sex, age, ethnicity, and household income. Additional details regarding the sample characteristics can be found in Table 1.

**Table 1:** Participant demographics (N = 106)

	M *(SD); N* (%)
Age	14.44 (1.69)
*Sex*	
Male	47 (52.80)
Female	42 (47.20)
*Race/ethnicity*	
White	15 (16.90)
Black	4 (4.50)
Hispanic/Latino	49 (55.10)
Asian	2 (2.20)
Native American	1 (1.10)
Middle Eastern	1 (1.10)
Other	2 (2.20)
Biracial/multiracial	15 (16.90)
*Annual household income*	
< $10,000	14 (15.90)
$10,000–$19,999	10 (11.40)
$20,000–$29,999	11 (12.50)
$30,000–$39,999	11 (12.50)
$40,000–$49,999	11 (12.50)
$50,000–$74,999	13 (14.80)
$75,000–$99,999	4 (4.50)
$100,000–$150,000	9 (10.20)
> $150,000	5 (5.70)

#### Emotional Eating

Emotional eating was assessed via The Emotional Eating Scale-Adapted for Children and Adolescents (EES-C; [28]), a 10-item, short-form of the original, 25-item EES-C measure. The short-form of the EES- C includes the 10 major eating-associated emotions from the original scale (i.e., jealousy, confusion, nervousness, anger, guilt, helplessness, feelings of not doing enough, sadness, loneliness, and disobedience) and was chosen to reduce questionnaire burden. In response to each of the aforementioned emotions, participants were asked to rate their desire to eat on a 5-point Likert scale, ranging from 1 (*No desire*) to 5 (*Very strong desire to eat*) with higher total scores indicative of a greater desire to eat in response to negative mood states. If participants did not experience a negative mood state, they were given a score of zero for that item. The original EES-C generates three subscales reflecting the urge to eat in response to anger, anxiety, and frustration; depressive symptoms; and feeling unsettled [49]. While the short-form version used in the present study does not have previously validated subscales, we calculated subscales using corresponding items and present these as exploratory analyses that provide a preliminary examination of how sleep and body dissatisfaction relate to eating in relation to more nuanced emotions. The original EES-C has demonstrated good internal consistency, temporal stability, and discriminant validity in adolescent samples [2] and the short-form has good internal consistency, construct validity, and high degree of overlapping variance with the original EES-C total score and subscale scores [28]. Reliability in our sample was good for the Emotional Eating Total subscale, α = 0.83, and acceptable for the adapted Angry/Anxious/Frustrated subscale, α = 0.71 and Feeling Depressed subscale, α = 0.73. As only one item comprised the Feeling Unsettled subscale using short-form items, reliability could not be calculated.

#### Body Dissatisfaction

Adolescent participants completed the Body Dissatisfaction Scale (BDS; [32]. The BDS presents nine computer-generated female and male body images in ascending order of body size, all of which are associated with a specific BMI ranging from underweight to obese. First, participants were asked to select the body figure they would most like to look like (i.e., ideal body) followed by the body they think is the most similar to their actual body shape (i.e., actual body). Participants’ body dissatisfaction score was calculated by subtracting the number associated with their actual body shape from the number associated with their ideal body shape, with higher scores indicating greater body dissatisfaction. The BDS for both the male and female versions exhibit good construct validity and test–retest reliability over a five-week period [32].

#### Sleep Difficulties

**Time-in-Bed.** Participants were asked to report their typical weekday bedtimes and wake times on an average school night and time-in-bed in minutes was calculated from responses to these items and used as a proxy for sleep duration. Time-in-bed is a commonly used proxy for sleep duration in adolescent sleep research and is associated with important outcomes (e.g., BMI; [18]) although it is limited in that it does not inherently account for important aspects of sleep duration such as sleep onset latency and night awakenings [34].

**Insomnia Severity.** Adolescents completed the Pediatric Insomnia Severity Index (PISI; [10], a 6-item, self-report measure of insomnia severity. Participants were asked to use the past week when answering questions related to how many nights per week they experience difficulty falling asleep, difficulty maintaining sleep, daytime sleepiness, and sleep duration. All items used a 6-point Likert scale, ranging from 0 (*0 nights per week*) to 5 (*7 nights per week*) with the exception of the average nightly sleep duration item which is scaled as 0 (*less than 5 h*), 1 (*5–7 h*), 2 (*7–8 h*), 3 (*8–9 h*), 4 (*9–11 h*) and 5 (*11–13 h*). Total scores range from 0 to 30, with higher scores representing greater insomnia severity. The PISI has been shown to have acceptable validity through correlation with other validated sleep measures (*r* = 0.42) and has also demonstrated high internal consistency (α = 0.80; [10]). Reliability in our sample was good for the PISI, α = 0.84.

### Statistical Analysis

First, descriptive statistics for demographics and primary variables were examined. Next, bivariate correlations between primary variables were run. Finally, to determine the relationship between sleep disruption (insomnia severity and time-in-bed), body dissatisfaction, and the interaction between sleep disruption and body dissatisfaction on emotional eating, a total of eight linear regression moderation analyses were run. Emotional eating was measured via the total score for primary analyses as well as through each of the three short-form adapated subscales for exploratory analyses: emotional eating in response to anxiety, anger, and frustration; depression; and feeling unsettled. For each analysis, the sleep variable was entered on the first step, participants’ body dissatisfaction score was entered on the second step, and the interaction between the sleep variable and body dissatisfaction was entered on the third step. Both the continuous insomnia severity and body dissatisfaction variables were mean-centered to enhance interpretation. All significant interactions were probed at high (+ 1 *SD*), moderate (mean), and low (− 1 *SD*) levels of body dissatisfaction, a standard and recommended approach used for significant moderation effects [25]. IBM Statistical Package for the Social Sciences (Version 28.0) software was used to conduct all analyses. An a priori power analysis was conducted using G*Power [22] with alpha equal to 0.05, 80% power, and three predictors in a linear regression model. The power analysis projected a need for 77 participants to detect a significant medium effect size (*f*^2^ = 0.15).

## Results

### Bivariate Analyses

Bivariate analyses were conducted to explore the relationships among the primary variables of interest. Less time-in-bed was related to greater body dissatisfaction (*r* =  − 0.23, *p* < 0.05) and greater insomnia severity was related to worse emotional eating across all adapted subscales – emotional eating total score (*r* = 0.28, *p* < 0.01), emotional eating in response to feeling anxiety, anger, and frustration (*r* = 0.27, *p* < 0.01), and emotional eating in response to feeling depressed (*r* = 0.27, *p* < 0.01)—with the exception of feeling unsettled (*p* > 0.05). Body dissatisfaction was not related to any aspect of emotional eating (*p* > 0.05). See Table 2 for detailed information about correlations and descriptives for primary variables.

**Table 2:** Descriptive statistics and bivariate correlations between primary variables

Primary variables	PISI	Time-in-bed		BDS	Total EES	EES_ AAF	EES_ DEP	EES_ US
Time-in-bed	.01		–					
BDS	− .01		− .23*	–				
Total EES	.28**		− .02	− .08	–			
EES_AAF	.27**		− .002	− .11	.93**	–		
EES_DEP	.27**		− .01	− .05	.88**	.67**	–	
EES_US	.02		− .13	.08	.54**	.44**	.39**	–
Mean(SD)	13.81(7.21)		501.39(90.96)	1.05(1.51)	9.32(8.41)	5.58(4.94)	3.34(3.72)	.40(.98)

PISI = pediatric insomnia severity index, BDS = body dissatisfaction scores, Total EES = total emotional eating scores, EES_AAF = emotional eating scores-anxiety/anger/frustration subscale, EES_DEP = emotional eating scores—depression subscale, EES_US = emotional eating scores-unsettled subscale. *p < .05. **p < .01

### Primary Moderator Analyses

#### Time-in-Bed and Total Emotional Eating Score

It was hypothesized that there would be a moderating effect of body dissatisfaction on the relationship between time-in-bed and emotional eating. Body dissatisfaction did not significantly moderate the relationship between time-in-bed and the emotional eating total score although it was approaching significance, *F*(3, 93) = 2.64, *p* = 0.054.

#### Insomnia Severity and Total Emotional Eating Score

It was hypothesized that there would be a moderating effect of body dissatisfaction on the relationship between insomnia severity and emotional eating total score. This hypothesis was not supported as body dissatisfaction did not significantly moderate the relationship between insomnia severity and overall emotional eating, *p* > 0.05. However, insomnia severity was found to be significantly associated with emotional eating total score within this model (*b* = 0.33, 95% CI [0.10, 0.56], *p* < 0.01) such that worse insomnia severity was associated with greater overall emotional eating.

### Exploratory Analyses

**Fig. 1:**
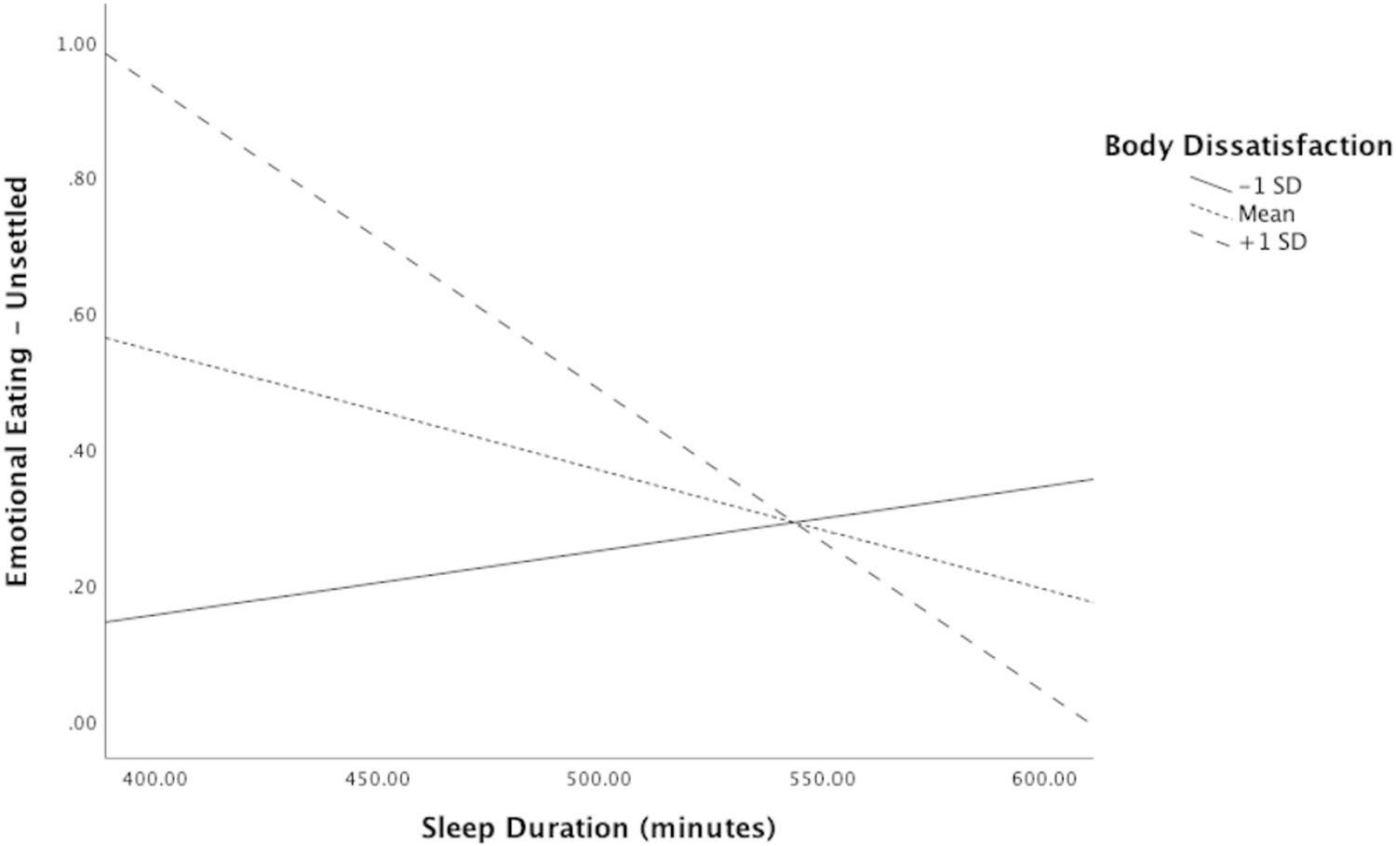
Model of the relationship between time-in-bed and emotional eating in response to feeling unsettled

**Fig. 2:**
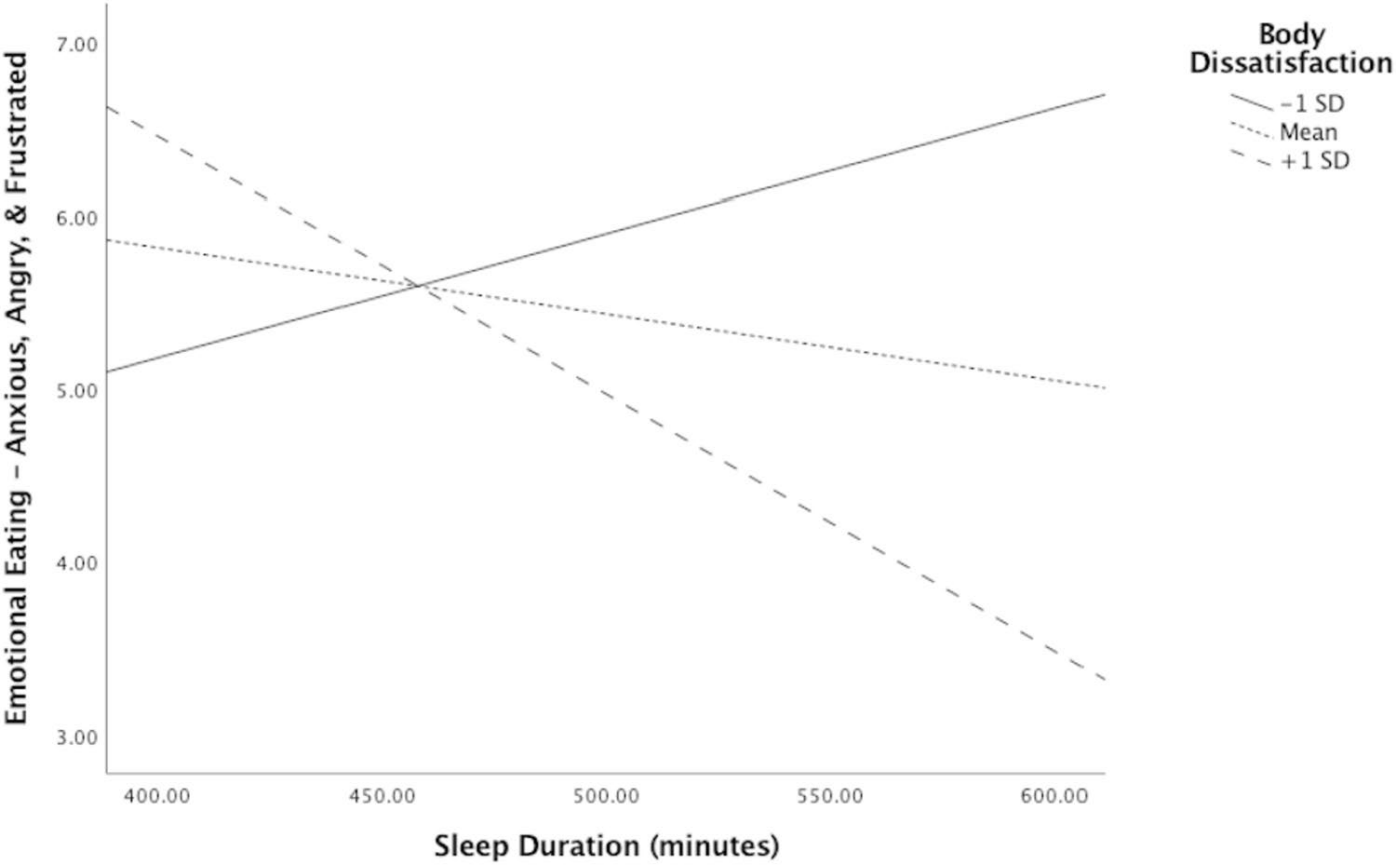
Model of the relationship between time-in-bed and emotional eating in response to feeling anxious, angry, and frustrated

In moderation analyses with time-in-bed, body dissatisfaction did not significantly moderate the relationship between time-in-bed and emotional eating in response to feeling depressed, *p* > 0.05. However, body dissatisfaction did significantly moderate the relationship between time-in-bed and emotional eating in response to feeling unsettled, *b* =  − 0.002, 95% CI [− 0.003, − 0.001], *p* < 0.005; see Fig. 1. The optimal linear combination of time-in-bed, body dissatisfaction, and the interaction between the two accounted for approximately 7.60% of the variance in emotional eating in response to feeling unsettled, *ΔR*^2^ = 0.09, *F*(3,93) = 3.63, *p* < 0.05. Among adolescents who reported less time-in-bed, those who sought to be thinner (i.e., positive body dissatisfaction score) reported significantly greater levels of emotional eating when feeling unsettled, *b* =  − 0.005, *t* =  − 2.89, *p* < 0.05. Those who had negligible body dissatisfaction (*b* =  − 0.002, *t* =  − 1.55, *p* > 0.05) as well as those who had negative body dissatisfaction (*b* = 0.001, *t* = 0.70, *p* > 0.05) reported nonsignificant levels of emotional eating in response to feeling unsettled. Similarly, body dissatisfaction significantly moderated the relationship between time-in-bed and emotional eating in response to anxiety, anger, and frustration, *b* =  − 0.01, 95% CI [− 0.01, − 0.001], *p* < 0.05 (see Fig. 2). The optimal linear combination of time-in-bed, body dissatisfaction, and the interaction between the two accounted for approximately 5.40% of the variance in emotional eating in response to anxiety, anger, and frustration, *ΔR*^2^ = 0.06,* F*(3,93) = 2.84, *p* < 0.05. Among adolescents who reported less time-in-bed, those who sought to be thinner (i.e., positive body dissatisfaction score) reported significantly greater levels of emotional eating when feeling angry, anxious, and frustrated, *b* =  − 0.01, *t* =  − 1.98, *p* = 0.05. Those who had negligible body dissatisfaction (*b* =  − 0.004, *t* =  − 0.69, *p* > 0.05) as well as those who had negative body dissatisfaction (*b* = 0.01, *t* = 1.09, *p* > 0.05) reported nonsignificant levels of emotional eating in response to feeling angry, anxious, and frustrated (Table 3).

**Table 3:** Results of full model multiple regression analyses associating emotional eating subscales and average time-in-bed, body dissatisfaction, and their interaction

	*R*^2^_adj_	*b*	β	*pr*^2^	*sr*^2^
*Total EES*	.05				
TIB		− .01	− .08	.01	.01
BDS		5.83*	1.09	.05	.05
TIB × BDS		− .01*	− 1.24	.07	.06
*EES-DEP*	.002				
TIB		− .002	− .05	.00	.00
BDS		1.63	.69	.02	.02
TIB × BDS		− .003	− .79	.03	.03
*EES-US*	.08				
TIB		− .002	− .15	.02	.02
BDS		.95**	1.44	.09	.09
TIB × BDS		− .002**	− 1.43	.09	.09
*EES-AAF*	.05				
TIB		− .004	− .07	.00	.00
BDS		3.25*	1.03	.05	.04
TIB × BDS		− .01*	− 1.21	.06	.06

BDS = body dissatisfaction; TIB = average time-in-bed. Total EES = total emotional eating scores, EES_AAF = emotional eating scores-anxiety/anger/frustration subscale, EES_DEP = emotional eating scores—depression subscale, EES_US = emotional eating scores-unsettled subscale. *p < .05. **p < .01

In regards to the models with insomnia severity, body dissatisfaction was not significantly associated with the emotional eating subscales for all three moderation analyses, *p*s > 0.05. However, insomnia severity was found to be significantly associated with emotional eating in response to feeling anxious, angry, and frustrated (*b* = 0.19, 95% CI [0.05, 0.32], *p* < 0.01) and emotional eating in response to depression (*b* = 0.14, 95% CI [0.04, 0.24], *p* < 0.01), but not with emotional eating in response to feeling unsettled, *p* > 0.05 (Table 4).

**Table 4:** Results of full model multiple regression analyses predicting emotional eating subscales from insomnia severity, body dissatisfaction, and their interaction

	*R*^2^_adj_	*b*	β	*pr*^2^	*sr*^2^
*Total EES*	.06				
INS		.33**	.28	.08	.08
BDS		− .46	− .08	.01	.01
INS × BDS		.07	.09	.01	.01
*EES-DEP*	.06				
INS		.19**	.27	.07	.07
BDS		− .38	− .12	.01	.01
INS × BDS		.04	.09	.01	.01
*EES-US*	.05				
INS		.14**	.27	.07	.07
BDS		− .12	− .05	.00	.00
INS × BDS		.02	.07	.00	.00
*EES-AAF*	.00				
INS		.00	.02	.00	.00
BDS		.05	.07	.01	.01
INS × BDS		.01	.15	.02	.02

BDS = body dissatisfaction; INS = insomnia severity. Total EES = total emotional eating scores, EES_AAF = emotional eating scores—anxiety/anger/frustration subscale, EES_DEP = emotional eating scores-depression subscale, EES_US = emotional eating scores-unsettled subscale. *p < .05. **p < .01

## Discussion

The present study examined the relationship among sleep disruption (defined as short time-in-bed or insomnia), body dissatisfaction, and emotional eating in adolescents presenting with sleep problems. It was found that less time-in-bed was related to a greater desire for thinness and insomnia severity was related to emotional eating in response to feelings of depression, anger, anxiety, and frustration in this sample. Moreover, findings suggested that body dissatisfaction moderates the relationship between shorter time-in-bed and certain aspects of emotional eating. Specifically, for teens who desire to be thinner, the relationship between less time-in-bed and the urge to emotionally eat was intensified – specifically in response to feeling unsettled as well as angry, anxious, and/or frustrated. Notably, the unsettled scale contained one item related to feeling disobedient which may be associated with externalizing behavior. These results are consistent with previous research finding a positive relationship between adolescents’ physiological vulnerabilities (i.e., less sleep) and emotional eating [42] while also shining light on a possible third variable at play in the relationship (i.e., body dissatisfaction). While previous research on young adult females (ages 18–22 years) has shown that higher levels of body image dissatisfaction were significantly associated with sleep maintenance difficulties and non-restorative sleep [46], the current study has replicated these findings in a adolescent sample with sleep problems. Futhermore, these findings are in line with past research that has posited increased cortisol is linked to disinhibited food intake in restrained eaters [54]. For those who desire thinness, and may try to restrict their eating, cortisol increasing emotions such as stress, anger, anxiety, and frustration are linked to increased eating, regardless of hunger. Decreased sleep only furthers their increased cortisol levels, giving further support to the current findings [24].

While several study hypotheses were supported, others were not. Specifically, time-in-bed was not directly associated with emotional eating in the absence of body dissatisfaction. Despite previous research showing that adolescents and children with short sleep duration tend to report higher food-related disinhibition and food-seeking behavior [7] as well as increased emotional eating [9], the current sample did not show the same pattern. It is possible that the current sample is not representative of the general population, as participants were presenting to a sleep study and some likely had other medical comorbidities.

Body dissatisfaction was also not directly associated with emotional eating within the examined adolescent population. We predicted such a correlation would exist among adolescents based on previous research suggesting that insomnia symptoms are significantly associated with certain aspects of body dissatisfaction, specifically negative cutaneous body image [1]. Additionally, other studies have shown that sleep-deprived adolescents are more likely to interpret neutral stimuli as negative [38] and engage in disinhibited eating. It is possible that body dissatisfaction in adolescents is not associated with emotional eating, but rather, is an additional consequence of disrupted sleep, thereby contributing to negative psychological and physiological consequences.

Overall, the findings that time-in-bed and body dissatisfaction are related and that dissatisfaction exacerbates the relationship between time-in-bed and emotional eating in response to feeling unsettled anxious, angry, and frustrated are consistent with prior research. Specifically, shorter sleep in adolescent populations is related to heightened sensitivity to the negative affect of others (which subsequently may influence body dissatisfaction), higher likelihood of experiencing negative emotions and reduced expression and feeling of positive emotions (which could impact emotional eating in response to these emotions), and more frequent shifts in the interpretation of neutral stimuli into negative [38].

With respect to prior research emphasizing the connection between body dissatisfaction and the manifestation of altered eating habits (i.e., emotional eating, restrained eating, binge eating), emotional eating habits may heighten the risk of developing a clinical eating disorder [56]. While the cross-sectional nature of the present study precludes speculation about directionality or causality, findings do support a relationship between insomnia severity and emotional eating and, further, less time-in-bed and a drive for thinness, even in the absence of an emotional eating response. Thus, it is possible that disrupted sleep may be a contributing factor to the onset or exacerbation of altered eating habits, although more research is needed to examine this hypothesis. These findings are particularly relevant to our adolescent sample, as the development of disordered eating has the highest prevalence in teens, particularly between the ages of 14 and 19 years [47]. The present findings contribute to prior research which has found that emotional stress, higher levels of body dissatisfaction, increased independence with regard to food choice, and poor sleep habits greatly enhance adolescents’ risk for maladaptive behavior patterns [40].

Prior studies examining sleep in patients with eating disorders have found that adolescents with anorexia nervosa and prolonged dietary restriction exhibit shorter sleep, greater sleep disruption, more awakenings, and lower sleep efficiency relative to age-matched controls [14, 37, 46]. Furthermore, examinations of sleep and bulimic behavior have shown that greater difficulty falling asleep and daytime sleepiness are linked to more severe bulimia symptoms [55]. While the mechanisms of these relationships are still unclear, decreased sleep and eating disorder symptoms have been seen to covary systematically, with a higher severity of sleep disruption associated with a higher severity of disordered eating symptoms [29]. As the present findings suggest that less time-in-bed associated with eating in reaction to feeling unsettled as well as feeling anxious, angry, and/or frustrated for some adolescents who seek to be thinner, it is possible that changes in eaitng behavior and body acceptance can be improved by addressing sleep. Specifically, cognitive behavioral therapy for insomnia (CBT-I) has been shown to be effective in treating sleep problems in adolescents [35] and could be integrated into disordered eating treatment. Other evidence-based treatments that may be considered include dialectical behavioral treatment (DBT) which has been shown to be effective in improving affective regulation and disordered eating behaviors when combined with CBT [6] while also including skills that may help stabilize sleep.

While the present study contributed to the understanding of poor sleep, body dissatisfaction, and emotional eating in adolescents with sleep concerns, limitations do exist. First, this study was cross-sectional and thus directionality of relationships cannot be determined. While it is possible that disrupted sleep may directly impact body dissatisfaction and/or emotional eating, it is likely that these relationships are bidirectional and body dissatisfaction and emotional eating could also impact sleep in adolescents. These constructs should be examined longitudinally across time and alternative analytic strategies (e.g., mediation) should be considered. Secondly, while the current study provides evidence that body dissatisfaction in the direction of desiring thinness significantly moderated the relationship between less time-in-bed and emotional eating in response to feeling anger, anxiety and frustration and feeling unsettled, the EES-C short form subscales used to capture emotional eating in response to specific emotions were adapted from the original subscales and not formally validated prior to use. Future studies may choose to use the EES-C full form in their analyses to ensure that the total and subscale scores are validated. Further, all data was self-reported by adolescents which may not be as reliable as objective measures and may also contribute to shared method variance. Further, adolescents reported on bedtime and wake time so that time-in-bed could be calculated as a proxy of sleep duration. However, this construct is limited in that it may be biased by reporting error and perceptions of sleep, which may not be correlated with actual sleep duration, and does not account for other important aspects of sleep such as night awakenings. Future studies may improve upon the current findings by using other reporters (e.g., parents), additional validated measures, and objective measures of sleep duration and insomnia severity such as actigraphy. Prior research examining relationships between sleep and eating among adolescents has successfully utilized actigraphy-measured sleep duration, providing a valuable objective measure of a difficult variable to accurately self-report [27, 39]. Additionally, the aim of the current study was to test the influence of body dissatisfaction on the relationship between sleep disruption and emotional eating; more specifically, whether body dissatisfaction strengthened or attenuated this relationship. However, gaining a clearer understanding of the underlying mechanisms that help explain the relationship between these variables would answer an interesting and important question. Thus, future researchers should use mediation models to examine how body dissatisfaction explains the relationship between sleep disruption and emotional eating. Futhermore, while study implications may inform practice related to disordered eating behaviors, participants were not screened for clinical eating disorders prior to participating and thus findings cannot be specifically generalized to this clinical population. Relatedly, all participants were recruited during an overnight sleep study and had a suspected clinical sleep disorder which may limit generalizability to other adolescent samples. Future research should control for organic sleep disorders and/or other physical and mental health comorbidities.

## Summary

The present study explored relationships among sleep difficulties, emotional eating, and body dissatisfaction among adolescents presenting with existing sleep concerns. Shorter sleep was related to a greater desire to be thin and more severe insomnia was related to most aspects of emotional eating. While shorter sleep was not bivariately associated with emotional eating across all youth, it was significant for adolescents desiring to be thin, suggesting adolescents with body dissatisfaction may be more vulnerable to the effects of short sleep. Given that adolescents are both at risk of short or disrupted sleep and are also the most at-risk age group for disordered eating onset, the way in which these related constructs (i.e., sleep, emotional eating, and body dissatisfaction) are related is relevant to both clinical practice and should be considered in future research.

## Data Availability

Data will be made available upon reasonable request.
